# When does cultural evolution become cumulative culture? A case study of humpback whale song

**DOI:** 10.1098/rstb.2020.0313

**Published:** 2022-01-31

**Authors:** Ellen C. Garland, Claire Garrigue, Michael J. Noad

**Affiliations:** ^1^ Centre for Social Learning and Cognitive Evolution, and Sea Mammal Research Unit, Scottish Oceans Institute, School of Biology, University of St. Andrews, St. Andrews, Fife KY16 8LB, UK; ^2^ UMR ENTROPIE, (IRD, Université de La Réunion, Université de la Nouvelle-Calédonie, IFREMER, CNRS, Laboratoire d'Excellence – CORAIL), 98848 Nouméa, New Caledonia; ^3^ Opération Cétacés, 98802 Nouméa, New Caledonia; ^4^ Cetacean Ecology and Acoustics Laboratory, School of Veterinary Science, University of Queensland, Gatton, Queensland 4343, Australia

**Keywords:** song, cultural evolution, cultural revolution, complexity, cetaceans, social learning

## Abstract

Culture presents a second inheritance system by which innovations can be transmitted between generations and among individuals. Some vocal behaviours present compelling examples of cultural evolution. Where modifications accumulate over time, such a process can become cumulative cultural evolution. The existence of cumulative cultural evolution in non-human animals is controversial. When physical products of such a process do not exist, modifications may not be clearly visible over time. Here, we investigate whether the constantly evolving songs of humpback whales (*Megaptera novaeangliae*) are indicative of cumulative cultural evolution. Using nine years of song data recorded from the New Caledonian humpback whale population, we quantified song evolution and complexity, and formally evaluated this process in light of criteria for cumulative cultural evolution. Song accumulates changes shown by an increase in complexity, but this process is punctuated by rapid loss of song material. While such changes tentatively satisfy the core criteria for cumulative cultural evolution, this claim hinges on the assumption that novel songs are preferred by females. While parsimonious, until such time as studies can link fitness benefits (reproductive success) to individual singers, any claims that humpback whale song evolution represents a form of cumulative cultural evolution may remain open to interpretation.

This article is part of a discussion meeting issue ‘The emergence of collective knowledge and cumulative culture in animals, humans and machines’.

## 1. Introduction

The field of animal culture has flourished over the past decade [1]; however, the very existence of culture in non-human animals has been controversial (e.g. [2,3]). Culture has played an important role in shaping human societies [4], from what we eat [5] through to the language(s) we speak (e.g. [6–8]). In essence, to be human is to be cultural. However, culture and its critical foundation, social learning, have now been documented across a wide variety of non-human animals (hereafter ‘animals’), from fruitflies (*Drosophila* sp.) through to cetaceans [9–11]. For example, controlled social diffusion experiments have demonstrated that chimpanzees (*Pan troglodytes*), bluehead wrasse (*Thalassoma bifasciatum*) and meerkats (*Suricata suricatta*) can socially learn solutions to tool-use problems, the location of mating sites and food acquisition techniques, respectively [12–14]. Other studies have demonstrated cultural transmission of tool use in bottlenose dolphins (*Tursiops* sp.) [15,16] and New Caledonian and Hawaiian crows (*Corvus moneduloides*, *C. hawaiiensis*) [17,18]. Where such experiments are not feasible (or ethical), the presence of cultural processes can be inferred through observed patterns of behavioural expression that are shared within populations but differ between populations [19–21]. Such studies, while not direct tests of social learning, provide robust, parsimonious inference for the presence of cultural processes [22].

To avoid confusion surrounding the use of the terms ‘social learning’ and ‘culture’, we define social learning as any learning process that is facilitated by the observation of, or interaction with, another animal or its products [9,22–25]. Social learning is essential for creating a culture. Here, we define culture as information or behaviours shared within a group, and acquired from conspecifics through some form of social learning [9,22,26]. Culture is able to act as a ‘second inheritance system’ whereby information is passed from generation to generation, creating stable cultures [11]. Behaviours or information can flow in multiple directions: vertically, from parent to offspring; obliquely, from a non-parent model to younger individuals; and finally, horizontally among peers [25].

Although the presence of culture and cultural behavioural traits is now well accepted, much contention surrounds the phenomenon of cumulative cultural evolution in animals [27,28]. Cumulative cultural evolution in essence is accumulation of modifications over time [27,29]. An individual or group invents, for example, a particular behaviour that is then modified (improved or refined) by a later user, and the new variation is again learnt socially and spreads through the population, creating a ‘ratchet effect’ [27,30,31]. This cumulative improvement in technological complexity through time combined with high-fidelity transmission have led to the pinnacle of human culture we observe today [28]. Cumulative cultural evolution has been suggested to occur in a variety of behavioural contexts from migratory routes to vocalizations in a handful of animal species including New Caledonian crows [32], homing pigeons (*Columba livia*) [33], big horn sheep (*Ovis canadensis*) [34], chimpanzees [35], zebra finches (*Taeniopygia guttata*) [36] and possibly killer whales (*Orcinus orca*) [37].

A classic example of cultural evolution is that of birdsong, where the patterns of songs change through time (e.g. [38]). Oscine songbirds learn their songs from an adult tutor (often fathers) [39], and in some species, there is continual learning throughout life allowing individuals to continually incorporate changes into their own song, and thus evolution of the song from season to season (e.g. corn bunting, *Emberiza (Miliaria) calandra* [40]; village indigo birds, *Vidua chalybeate* [41]). In village indigobirds and cowbirds (*Molothrus ater ater*) [41,42], males will copy the song of the most successful breeding male. Further, a three decade study of the cultural evolution in the savannah sparrow (*Passerculus sandwichensis*) indicated that parts of their song, including song variants, were associated with reproductive success [38]. Part of the cumulative cultural evolution debate, particularly involving birdsong [27], is the distinction between stochastic processes such as cultural drift, and cultural evolution. Song characteristics can change randomly through time, such as in the chowchilla (*Orthonyx spaldingii*) [43] and chaffinches (*Fringilla coelebs*) [44]; such drift represents a fitness-neutral learned behaviour and is consequently considered non-cumulative cultural evolution (as per [27]). By contrast, if cultural evolution confers some fitness advantage, such as an association between reproductive success and song, then this lends itself to cumulative cultural evolution (e.g. zebra finch song).

Given the historical debate surrounding cumulative cultural evolution (where reports of cumulative culture in animals are refuted as subjective, circumstantial, or ‘simple’ [28,45]), a set of core and extended criteria were suggested by [27] to allow evaluation of the phenomenon regardless of the species (human or otherwise) involved. The four core criteria include [27]:

(1) *Introduction* of behavioural variation through either the modification of an existing behaviour or emergence of an entirely new behaviour. This can occur through behavioural novelty, random copying errors, or other stochastic processes.
(2) *Transmission* of the behavioural variant via social learning.
(3) *Improvement* or enhancement of some measure of ‘performance’ (i.e. the desired characteristics of the socially learnt trait are maximized). This can be a proxy for inclusive fitness (direct or indirect reproductive success), ‘cultural fitness’ (indirect proxy, e.g. wealth or social status), aesthetic attractiveness, etc.
(4) *Repetition* over time of innovation and social learning to generate sequential improvement in performance.

These criteria are particularly suited to evaluation of a single behavioural trait. With arguably one of the most complex acoustic displays in the animal kingdom, humpback whale (*Megaptera novaeangliae*) song provides a robust test of cumulative cultural evolution of vocal displays.

**Figure 1.**
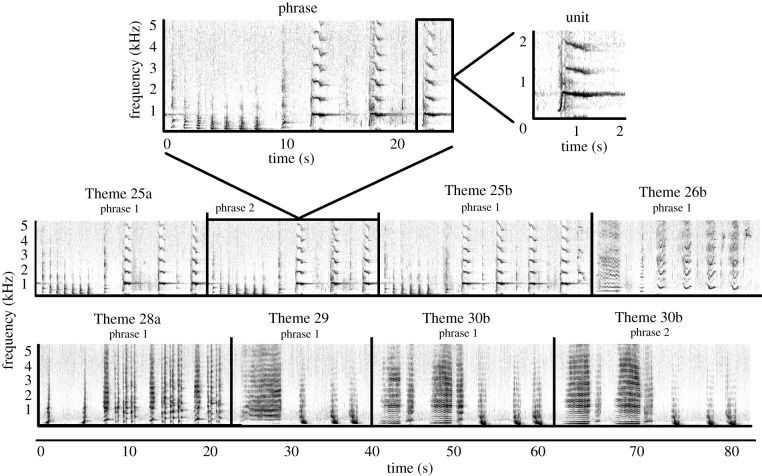
Spectrograms illustrating the hierarchical structure of humpback whale song. A single unit (trumpet) and a single phrase from Theme 25a are shown in the top panel. Theme 25a units from the single phrase in the top panel are as follows: short ascending moan, grunt, grunt, grunt, grunt, grunt, grunt, short ascending moan, trumpet, squeak, trumpet, squeak, trumpet. The repetition of phrases and the sequential singing of themes are shown in each of the subsequent panels. Spectrograms were 2048 point FFT, Hann window, 31 Hz resolution, and 75% overlap, generated in Raven Pro 1.4. Reprinted with permission from Garland *et al*. [48]. The devil is in the detail: quantifying vocal variation in a complex, multi-levelled, and rapidly evolving display. *J. Acoust. Soc. Am.* **142**, 460–472 [48]. Copyright 2017, Acoustic Society of America.

Humpback whale song is long, complex, repetitive and structured in a nested hierarchy [46,47]. Sound ‘units’ are sung in a stereotyped ‘phrase’, with repetition of the phrase comprising a ‘theme’ [46] (figure 1). Multiple different themes sung in a stereotyped sequence form a ‘song’ [46], and finally, different ‘song types’ are composed of a different suite of themes [20] (electronic supplementary material, figure S1a,b). Only mature males sing [50] and, within a population, most males conform to the current song arrangement at any point in time, demonstrating strong cultural conformity, but the song is also constantly evolving [51,52]. Songs evolve continually each year (particularly during the winter breeding season) at all levels within the song hierarchy: units can be added, split or deleted, as can entire themes [51,53]. This constant, gradual change within a season results in turnover of themes that progressively leads to a different song after a few years [52]. At the upper extreme, the entire song arrangement can be rapidly replaced, termed a ‘song revolution’ [54]. This occurs when a song type from a neighbouring population is introduced and rapidly replaces the existing song type [20,54]. This wholesale cultural change is very striking and provides a clear demarcation of evolutionary progression. Multiple song revolutions have spread across the South Pacific region from east Australia eastward across to French Polynesia, each taking approximately two years [20]. Recent work has highlighted an increase in song complexity (here measured as number of units in a song as well as diversity of unit types and unit arrangements; see §2c) as songs evolve and an abrupt decrease in complexity when a song revolution occurs [55]. The authors suggest that the introduction of completely novel material represents an upper limit to song learning, as an entire song must be rapidly learnt, not just a few new components. The increase in complexity during evolutionary periods has been interpreted by others [1,45] as anecdotal evidence for cumulative cultural evolution in humpback whales. These authors suggest that an increase in complexity illustrates the collective contributions of different animals’ innovations to cumulative culture change [45], by linking individual embellishment with complexity, despite [55] suggesting caution in such an interpretation. Finally, the building blocks to allow for cumulative cultural evolution in birdsong are explored by [56]; the structural components they identify (i.e. sound units, sequences of sounds) are the same as those present in humpback whale song structure, providing support for the potential for cumulative cultural evolution of multiple animal songs.

Here, we explicitly investigate whether humpback whale song evolution meets the above four criteria to qualify as cumulative cultural evolution. Controlled learning experiments are currently unfeasible in this species; therefore, we examine the products of cultural evolution without explicitly testing learning. Recent agent-based models that explicitly test social learning and cultural transmission of humpback song provide strong evidence for these underlying cultural processes [57,58]. Here, we quantify nine years of song evolution within a single population (New Caledonia) to understand changes in song content and complexity through time, and whether such changes meet the four core criteria for cumulative cultural evolution [27]. As with its neighbouring population, east Australia, to which the complexity method was previously applied [55], New Caledonia incorporates the phenomenon of ‘cultural revolutions’ to provide a clear, although extreme, introduction of behavioural novelty.

## 2. Methods

### (a) Field site and song recording

Humpback whale songs were recorded in the Southern Lagoon in New Caledonia from 1998 to 2006. The New Caledonian population is a small (351–772 individuals), genetically distinct population of humpback whales that breeds in the lagoons and seamounts surrounding New Caledonia, in the western South Pacific [59–62]. Recordings were made using a Sony DAT TCDD100 recorder and a single hydrophone (recorded digitally but then transferred to computer by digital to analogue conversion followed by re-digitizing at 44.1 kHz and 16 bit). Some singers in this study were not identified; this occurred as they were not sighted, and/or a photo-ID (or genetic) sample was not possible. Each song recording was treated as a separate singer unless singer ID information was present. All recordings were taken on different days except for two recordings (in 1999), which were separated by 2 h.

### (b) Acoustic analysis

Previous analyses have identified and quantified the five song types and 47 themes grouped into three song lineages (Black, Blue and Red; see electronic supplementary material, figure S1) present in New Caledonia from 1998 to 2006 [20,63–65] (table 1). Briefly, songs were transcribed by a human classifier (E.C.G.) into sequences of sound units based on the aural and visual characteristics of the unit types. To ensure these qualitative unit classifications were robust and repeatable at this base level of the song hierarchy, unit classifications were checked for consistency using discriminant function analysis with cross-validation (80% agreement in classification) and classification and regression tree analyses (88% agreement in classification) (see electronic supplementary information S1 and [20,63]). Unit sequences were then assigned to themes (labelled 1–47) and checked for consistent classification using a naive observer test, with greater than 94% agreement in classification [20,67]. Songs, which comprised the typical sequence of themes sung, were quantitatively assigned to song types (each with an arbitrary colour name) [63,64]. For each recording, all songs were included to increase the sample size. Each song represents a sample of what was being sung at that point in time in that year. In total, five song types containing 47 themes were present in New Caledonia from 1998 to 2006 (*n* = 46 singers, *n* = 214 songs). Data from 2004 contained two song types: the Blue song type was recorded from one singer at the start of the season while all other recordings that year were of the Dark Red revolutionary song type. Each song type in 2004 was analysed separately (table 1). All other years had only a single song type so all recordings were pooled together by year (table 1).

**Table 1.** Summary of data included in the song complexity analysis. Song measures: number of themes per song, number of unit types per song and duration of each song in seconds.

year	song type	# singers	# songs [# songs per singer]	themes present^a^	song measures (mean ± s.d.)		
					#themes	#unit types	duration(s)
1998	Black	5	28 [5,5,6,6,6]	6a,6b,7a,7b,8a,8b,9a,9b,10a	4.11 ± 2.44	11.86 ± 7.64	288.61 ± 127.79
1999	Black	6	21 [2,3,3,4,4,5]	6a,6b,7a,7b,8a,8b,9a,9b,10a,10b	7.10 ± 0.94	24.43 ± 4.79	511.62 ± 191.50
2000	Black	5	21 [2,3,3,5,8]	6a,7a,7b,8b,9a,9b,10a,10b,11,12,13,15a,15b	5.71 ± 2.88	22.81 ± 13.46	386.33 ± 231.70
2001	Dark blue	6	29 [3,4,4,6,6,6]	17a,17b,18,19,20,21,22	5.21 ± 1.61	20.41 ± 6.58	402.14 ± 173.68
2002	Blue	4	16 [2,2,5,7]	23,25a,25b,26a,26b,27,28b,29,30b	6.06 ± 0.85	20.81 ± 3.53	514.69 ± 169.18
2003	Blue	7	28 [1,1,3,3,3,8,9]	23,24,25a,25b,26b,27,28a,28b,29,30a,30b	8.82 ± 0.94	29.75 ± 4.43	616.04 ± 143.42
2004 a	Blue	1	5	25b,26b,27,28a,29,30a,30b	6.40 ± 0.55	23.40 ± 3.44	433.40 ± 157.27
2004 b	Dark red	3	23 [7,8,8]	31,32,33,34,36,37a,37b	4.39 ± 1.37	11.04 ± 4.14	266.35 ± 106.12
2005	Dark red	6	28 [3,4,4,5,6,6]	31,32,33,34,35,36,37a,37b	4.93 ± 1.02	18.21 ± 3.56	379.04 ± 157.31
2006	Light red	3	15 [3,6,6]	38,39,40,41,43	4.60 ± 0.51	25.00 ± 4.41	321.93 ± 129.07
total	5 song types	46	214	47 themes	—	—	—

^a^Themes were identified in multiple, previous studies [20,48,49,58,63–66].

### (c) Quantifying song evolution through complexity

Songs progressively evolve at all levels within the song hierarchy. To represent changes at multiple levels in the song at once, we computed song complexity scores. The scores incorporate multiple arrangement features in a singular measure to quantify changes in song complexity over time. We calculated humpback whale song complexity scores per song for each year following [55], which was based on scores calculated for song complexity in zebra finch [68,69]. In [55], three measures of song complexity were calculated: one at the theme level, one at the song level and one that combined all variables. The song-level variables included the number of unit types per song, the number of units per song and the duration of each song. The theme-level variables included the mean phrase duration per song, the number of themes per song and the mean individual theme complexity (calculated as a complexity score: number of unit types per phrase, theme duration and number of units for each phrase). All three measures (theme-level, song-level and all variables together) produced the same pattern (i.e. result) regardless of the ‘level’ of analysis [55], suggesting that song-level variables also capture the theme-level differences in unit type and number.

Here, we initially measured the four following song variables: number of units per song, number of unit types per song, duration (s) of each song and number of themes present per song. This suite of variables combined all song-level variables included in [55] and one of the three theme-level variables. Counting the number of units in the songs of 13 of the singers, however, was complicated by attenuation and resultant inaudibility of the song when the singer surfaced to breathe, a well-documented phenomenon. This did not affect assessment of the number of unit types used, number of themes, or song duration. Therefore, to maximize sample size, we removed the variable ‘number of units per song’ from the analysis. This resulted in three variables being included in the complexity scores: number of unit types per song, duration (s) of each song and number of themes present per song (*n* = 214 songs from 46 singers). To ensure patterns were robust, complexity scores including all four variables (*n* = 118 songs from 33 singers) were calculated. Results of the four-variable analysis are presented as electronic supplementary material, S1 but will not be discussed further.

**Figure 2.**
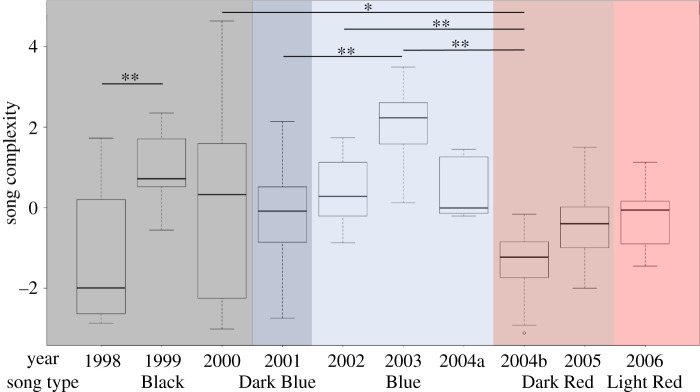
Song complexity through time including three song lineages—Black, Blue and Red. Complexity scores were computed from three song measures: # themes, # unit types and duration of each song. Box plots represent all songs in each year to show the variability in scores per year. 2004 had two song types present: the Blue song type was recorded at the start of the season (2004a: Blue box plot); all other recordings that year were of the Dark Red revolutionary song type (2004b: Dark Red box plot). Statistically significant (*p < 0.05, **p < 0.01) complexity scores (Kruskal–Wallis with Bonferonni correction for multiple comparisons) not included in the figure for ease of viewing include (electronic supplementary material, table S1): 1998 versus 2002*, 1998 versus 2003**, 1999 versus 2004b**, 1999 versus 2005*, 2000 versus 2003**, 2003 versus 2005** and 2003 versus 2006**.

The relationships among the three variables were checked using a Pearsons correlation test in R (v. 3.5.3); all variables were strongly correlated (see §3). Following [55,68,69], we conducted an unrotated principal components analysis (PCA) in R using the *princomp* function and extracted the first principal component for each song as the ‘song complexity score’. We chose to conduct the analysis using all available songs instead of a single representative song from each singer to capture the variability both within and between singers and ensure the patterns were robust. We acknowledge this leads to overrepresentation of individuals for which we had more data (table 1). Changes in complexity scores through time and how these related to periods of song evolution and revolution are presented as box plots (figure 2). To assess if the complexity scores were significantly different among years, a non-parametric Kruskal–Wallis test (and *post hoc* Bonferonni test for multiple comparisons) was conducted in R. Finally, to test whether song complexity significantly increased each year within a song lineage, a linear regression was conducted in R (see electronic supplementary material, S1 for further information).

## 3. Results

All three song measures were positively correlated. The number of themes was positively correlated with the number of unit types (*r* = 0.823, *p* < 0.001), and the number of themes was also positively correlated with duration of each song (*r* = 0.723, *p* < 0.001). Finally, the number of unit types was positively correlated with the duration of each song (*r* = 0.666, *p* < 0.001). The PCA of the three song measures per singer resulted in a single principal component (PC1) that explained 82.57% of the variance with an eigenvalue of 1.732. The unrotated component loadings on PC1 were 0.582 for the number of unit types per song, 0.554 for the duration of each song, and 0.596 for the number of themes present per song. The scores for PC1 for each song were extracted and represent the ‘song complexity score’.

**Figure 3.**
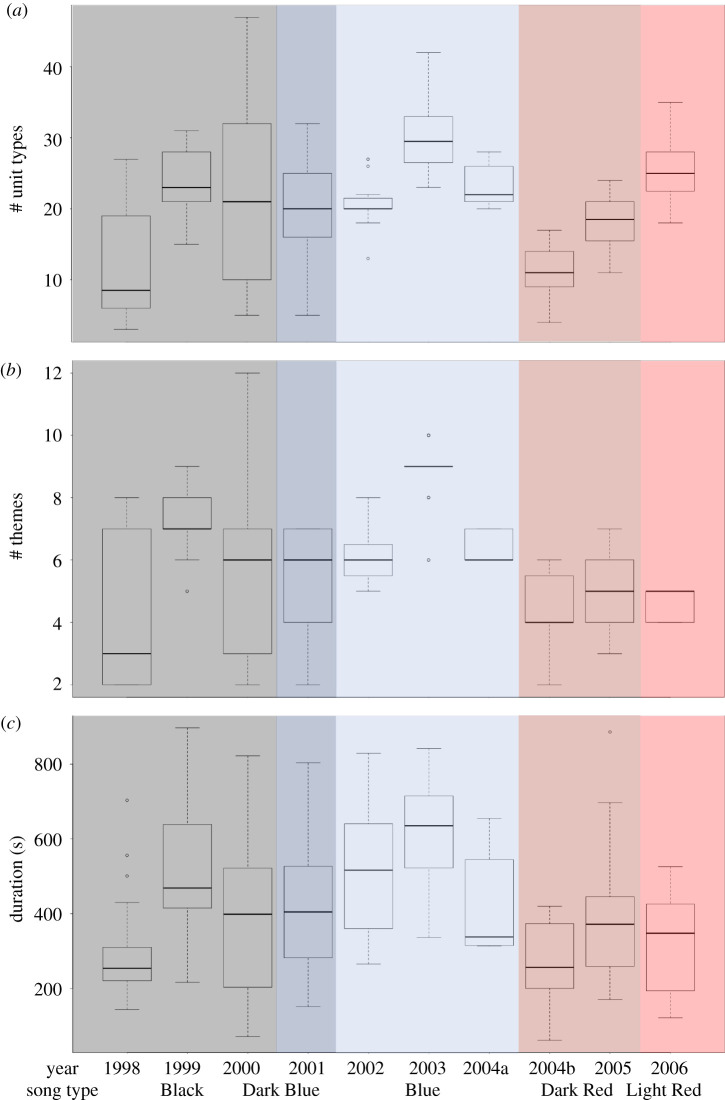
Box plots of (*a*) # unit types per year, (*b*) # themes per year, and (*c*) song duration (s) per year. Plots represent all songs in each year to show the variability per year (table 1). 2004 had two song types present: the Blue song type was recorded at the start of the season (2004a: Blue box plot); all other recordings that year were of the Dark Red revolutionary song type (2004b: Dark Red box plot).

A quasi-sinusoidal pattern was evident from the changes in complexity scores through time in songs (figure 2). Complexity scores were significantly different among years (Kruskal–Wallis, χ^2^ = 101.91, d.f. = 9, *p* < 0.001; electronic supplementary material, table S1), and song complexity significantly increased each year within song lineages (Adj *R*^2^ = 0.578, *F*-statistic = 11.98, d.f = 7, *p* = 0.011; electronic supplementary material, figure S2). Increases in complexity corresponded to periods of song evolution, while decreases in complexity matched time periods when song revolutions occurred. During evolutionary periods, complexity changes were best captured by the number of unit types and number of themes present. As unit types increased (figure 3 and table 1), complexity scores increased. For example, the Blue song type increased the number of unit types concurrently with increased themes sung per song (table 1 and figure 3*a,b*). The *post hoc* Bonferroni analysis indicated song complexity significantly increased during the Blue song lineage and the first two years of the Black song lineage (electronic supplementary material, table S1 and figure 2). Where songs were characterized through turnover of themes during evolution (addition of new themes and deletion of old themes), this reduced complexity, although not significantly (2000 Black and 2004a Blue; figure 2; electronic supplementary material, table S1). In 2000, the Black song type was evolving into what would become the Grey song type [20]. This occurred by adding themes (11,12,13,15a,15b) while starting to drop older Black themes, singing shorter songs, and including fewer themes per song. Finally, in all cases, just prior to a new song lineage being introduced (a song revolution) the existing song shows a slight but non-significant decrease in complexity (figure 2; electronic supplementary material, table S1), while the new revolutionary song may have significantly lower complexity (2003 Blue to 2004b–2006 Red; figure 2; electronic supplementary material, table S1).

## 4. Discussion

Song complexity within the New Caledonian humpback whale population changed significantly in a quasi-sinusoidal manner mirroring periods of song evolution and rapid revolution over nine years. This reinforces the findings in [55] where similar cyclical patterns were found in the song of the neighbouring east Australian humpback whale population. Humpback whale song clearly goes through periods of song evolution where themes, units and unit types are added with a corresponding increase in the complexity of songs. This is punctuated with completely novel songs (i.e. from a different song lineage; electronic supplementary material, figure S1) appearing within these populations and replacing the existing song (a ‘revolution’). All song revolutions included in this analysis were traced to come from the east Australian population [20]. While some of the increases in complexity during evolutions and decreases when a revolution occurred were significant (figure 2), this was in part due to applying a conservative *post hoc* analysis (see electronic supplementary material, table S1 for an alternative *post hoc* test). Below we explore whether humpback whale song evolution meets the four criteria—*introduction*, *transmission*, *improvement* and *repetition*—to qualify as cumulative cultural evolution [27].

New song material is *introduced* as the song clearly undergoes modifications to its existing arrangement at all levels within the song hierarchy (i.e. evolution) and periodically through the introduction of an entirely new song (i.e. revolution). Many previous studies from all ocean-basins have documented progressive song evolution through the turnover in units, themes and unit arrangements (e.g. North Pacific [70,71], North Atlantic [52,72], Africa [73], South Pacific [20,74]); in some cases at the decadal scale. Recent work employing agent-based models to understand the mechanism(s) driving humpback song evolution suggests that simple production errors coupled with a bias for novelty mirrored empirical data of song change [57]. Therefore, production errors, learning errors, deliberate innovation or a combination of the three may be causing the gradual evolution of the song [75]. We, along with other authors, hypothesize there is a strong sexually selected drive for novelty in humpback whale song that may be underlying the system [54,70]. If females were preferentially mating with males displaying novelty, whether in the form of small, evolutionary changes or large, revolutionary changes, then this would create a runaway system with little conformity. The paradox of humpback whale song is that it appears to concurrently display a conformist bias along with constant, sexually selected novelty bias, which results in a display that is constantly changing but conformist at any point in time. This has been termed ‘constrained novelty’ [76], where males will incorporate novelty into their songs while not individually diverging so that all songs are different. Future studies that investigate whether females preferentially mate with males singing novel songs or more complex songs are needed.

It is unclear whether the song is a conglomeration of many small changes from many males or if specific males are driving this change. In some songbirds, such as the village indigobird and cowbirds [41,42], males will copy the song of the most successful breeding male thus displaying a model bias potentially based on reproductive prestige. Long-term studies of the cultural evolution of savannah sparrow song further indicate that parts of their song, including song variants, were associated with reproductive success [38]. In humans, the role of prestige has been investigated in online, collaborative programming tournaments [77] where code can be copied from successful human computer programmers. These tournaments suggest an important role for prestige in the transfer of information to create cumulative cultural knowledge, where solutions to computer problems are sequentially improved by copying and innovation [77]. These ‘leaders’ (in solutions) exerted more influence than ‘non-leaders' on the patterns of solutions and thus the sequential improvement of this cumulative cultural evolution experiment [77]. This framework of ‘leaders' and ‘non-leaders' of sequential change provides an exciting future area for humpback song research in regard to assessing its place within the cumulative cultural evolution paradigm.

The behavioural variant—the song type—is *transmitted* among individuals and subsequently populations. Clear evidence of complete song types appearing and rapidly replacing the existing song in its entirety within a population has been repeatedly shown across the South Pacific region [20,49,54,55,58,64,66,67,78,79]. As controlled social learning experiments are currently unfeasible in this species, agent-based models that explicitly test social learning and cultural transmission of humpback song at the individual level have provided compelling evidence for these underlying cultural processes [57,58]. Extending this work to a global scale, cultural evolution models of song transmission suggest that simple learning rules can create population-level emergent properties where low levels of mutation in combination with rare population interactions match empirical song sharing patterns in the South Pacific, including the distinctive west to east pattern of revolutions [80]. This directional transmission appears driven by differences in population sizes, as hypothesized by [20,75]: songs spread from large to small populations [80]. Recent evidence from white-throated sparrow (*Zonotrichia albicolis*) song has highlighted a similar pattern of west to east song transmission across Canada, but with a far slower spread (a few decades versus two years) [81]. As hypothesized for humpback song transmission (termed the ‘novelty threshold hypothesis’ [76]), [81] suggest that a critical number of males were required to adopt the new song variant before the cultural spread became exponential.

The most difficult and controversial criterion to address here is that of *improvement*. Two major concepts are apparent from song and complexity analyses: the cyclical nature of complexity (figure 2 and [55]) and the well-established way in which song constantly changes (e.g. [52]). First, songs increase in complexity through time, but this is punctuated with loss of complexity as a completely novel song is introduced. While some types of cumulative culture are characterized by increasing complexity (e.g. horse and cart becoming the motor car, simple telephones evolving into smart phones) other types, such as fashion, do not. Complexity can therefore change in both directions, and this may be tied to the underlying driver of change (e.g. aesthetic attractiveness, efficiency, change for the sake of change, etc. [82,83]). Such turnover in song material is analogous to change in human fashion, which appears arbitrary and linked to current aesthetics and model bias (i.e. cultural fitness). One could argue that women's fashion in the Victorian era was far more complex than in the 2000s, but there has clearly been an improvement in functionality. For example, the (r)evolution of fashion by Coco Chanel in the early twentieth century with high couture clothes designed for comfort, practicality and simplicity clearly links fashion to aesthetic attractiveness and efficiency without increased complexity being a necessity.

The second concept is the constantly changing patterns of the song [51,53]. Given that revolutionary songs introduce a large amount of novel material that is rapidly learnt, [55] suggests that the lower complexity of revolutionary songs may be due to the whales only being able to learn a certain amount over a given period, and this may represent an upper limit to song learning. The ability to rapidly learn novel material and/or more complex songs may be indicative of the ‘cognitive capacity hypothesis’ [55], where complex songs and the ability to rapidly learn them may signal more developed cognitive abilities [84] that in turn may be sexually selected by females [68]. But if this is the case, then the cultural ‘artefact’—the song—may not in itself be particularly meaningful, rather it is the ability of the singer to adopt novelty and change their songs that is important, both in terms of sexual selection and *improvement*. A disconnect emerges between the content of the song, which may be arbitrary, and the ability of the singer to rapidly incorporate changes. In human society, being ‘fashionable’ has little to do with the utility of the actual clothing (e.g. improving survival), it is *being* fashionable that is ‘attractive’. Demonstrating the ability to identify and rapidly adopt new fashion trends shows superior social learning and cognitive abilities that increase a wearer's cultural fitness. Similarly, rapidly adopting changes to the song (i.e. artefact) might increase the reproductive potential of a male humpback whale. Studies investigating reproductive success of singers at the vanguard of song changes are needed to confirm this hypothesis.

If we interpret the criteria for *improvement* as the improvement of the aesthetic attractiveness of song to females, given the assumption that females should prefer more novel songs, then this criterion would be satisfied as the males are improving their fitness by changing their songs. Such a system seems highly plausible given the observed uptake of novel humpback whale songs within a population [20,76] in combination with the female-driven sexual selection [54,70] hypothesized above, and observed in some song birds [36]. Furthermore, such logic and acceptance of the female preference assumption is applied by [27] (electronic supplementary material, table S3) to tentatively conclude that song learning in zebra finch [36] fulfils the core criteria for cumulative cultural evolution.

Future studies, although extremely challenging in humpback whales, linking individually identified males with song recordings and their reproductive success will be helpful to confirm this criterion. However, assessing whether a humpback whale finds a song ‘pretty’ (i.e. aesthetically pleasing, which might increase cultural fitness) as opposed to ‘attractive’ to females (i.e. leading to direct or indirect reproductive success) is currently outside the scope of behavioural experiments. This makes teasing apart the cultural process from those of sexual selection difficult. Therefore, the current state of evidence to satisfy the criterion of *improvement* is ambiguous: if we interpret this as allowing the assumption of an aesthetically attractive characteristic of song to females, then it is met, but if we apply a strict interpretation requiring a proven link to reproductive success, then it is not met.

Finally, there is clear evidence that innovation and social learning are *repeated* over time and in multiple populations to generate changes to the song [20,52,57,80]. Song changes in a unidirectional manner over multiple years in a population at all levels in the song hierarchy [52,53,85]; males must repeatedly learn these changes and incorporate them into their own song to maintain the observed conformity. The increase in song complexity demonstrated above within a population (figure 2) provides a clear ‘ratcheting’ up of complexity as songs evolve. This is further complemented by the rapid, repeated and regular transmission of different song types (lineages) across the South Pacific providing a striking example of population-wide song transmission and learning at the ocean basin scale [20]. During song revolutions, a few recordings of combined or hybridized (‘old’ and ‘new’) songs have been identified that are hypothesized to be instances of a whale in the process of learning a new song [49]. Hybrid songs were segmented into themes and the position where singers transitioned from singing an ‘old’ to ‘new’ song theme was not random; singers followed a ‘switch when similar rule’, where similarity in unit type and arrangement was highest between the song types allowing a smooth transition between the songs [49]. As songs can be combined in predictable ways based on structural patterns and the display is learned as segments similar to birdsong and human language acquisition (e.g. [86–92]), it may provide a comparative perspective on the evolution of human language. Increased structure and ease of learning have both been identified in human language iterative learning experiments as important in the evolution of language [6], and highlighted in [82] where ease of learning is central to the transmission of complex behaviours. Future studies investigating whether similar dynamics emerge in iterative models of humpback whale song evolution may shed light into the origins of complex communication. Finally, we have not explored the extended criteria for cumulative cultural evolution suggested by [27]: functional dependence of multiple cultural traits, diversification, recombination and cultural exaptation. The potential for diversification or recombination of song lineages may present an interesting starting point for exploring these extended criteria in humpback whales.

## 5. Conclusion

Here, we have shown that song complexity changes in a quasi-sinusoidal manner mirroring periods of song evolution and rapid revolution over nine years within the New Caledonian humpback whale population. Song accumulates changes shown by an increase in complexity, but this process is punctuated by rapid loss of song material. We have robustly met three of the four core criteria for cumulative cultural evolution—*introduction*, *transmission* and *repetition*—but it is open to interpretation whether we have meet the criteria for *improvement* [27]. Until studies can link reproductive success to individual singers, any claims that humpback whale song evolution represents a form of cumulative cultural evolution will remain tentative and potentially contentious. The emerging parallels in the investigation (e.g. agent-based and cultural evolution models) and understanding of cultural processes in birdsong, whale song and human language provide a rich avenue for future comparative research.

## Data accessibility

Song data from 1998 to 2006 can be found in [20,47,50,51]. All measurements for both complexity analyses are provided in electronic supplementary material, S2 [93]. Please seek prior written permission from E.C.G. to reuse data in any form except to confirm the results of this study.
